# Supplementary material for: Liquid-Modulated Photothermal Phenomena in Porous Silicon Nanostructures Studied by μ-Raman Spectroscopy
Source: Nanomaterials (Basel). 2023 Jan 11;13(2):310. doi: 10.3390/nano13020310 (PMC9867246; doi:10.3390/nano13020310)
Supplement: Supplementary file 1 [file nanomaterials-13-00310-s001.zip › nanomaterials-2132358-supplementary.pdf]

Liquid-Modulated Photothermal Phenomena in Porous Silicon Nanostructures Studied by μ-Raman Spectroscopy

Oksana Makukha [1], Ivan Lysenko [2] and Ali Belarouci [1,*]

[1] Lyon Institute of Nanotechnology, UMR 5270, INSA de Lyon, 69100 Villeurbanne, France
[2] Physics Department, Taras Shevchenko National University of Kyiv, 01033 Kyiv, Ukraine
* Correspondence: ali.belarouci@ec-lyon.fr

1. Thicknesses of porous Si layers

Thicknesses of porous Si layers were estimated by SEM. A typical cross sectional image of porous Si sample is presented in Figure S1.

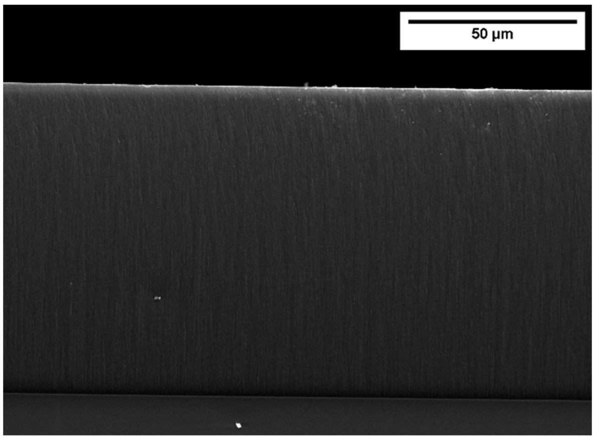

Figure S1. Cross-sectional SEM image of porous Si layer.

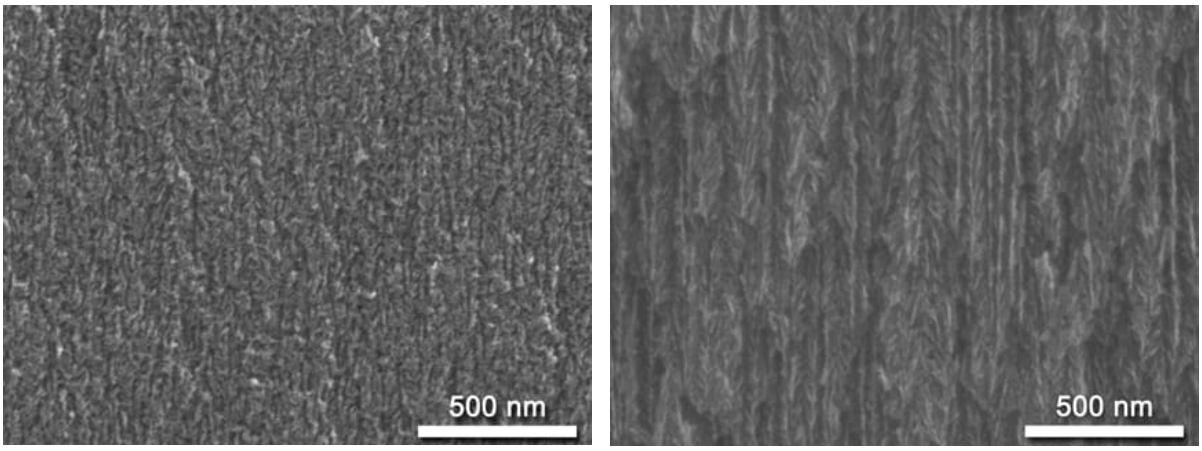

(a) (b)

Figure S2. Cross-sectional SEM image of porous Si layer of (**a**) sample with low porosity (≈ 40%) and (**b**) high porosity (≈ 80%).

2. Porosity and hexadecane filling factor calculation

The reflectance spectra of porous Si layers were collected in the 1400–1700 nm wavelength range.

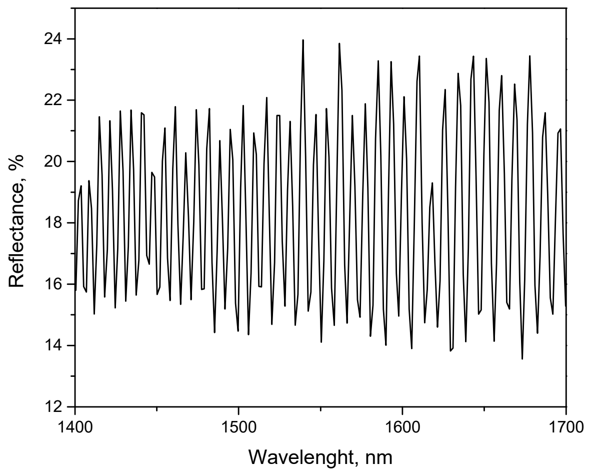

Figure S3. Reflectance IR spectrum of porous Si sample with 78,7% porosity.

2.1. Determination of refractive index of porous Si layer

For each interference maxima position (minima in reflectance scale), the following equation is satisfied:

$$N \cdot \lambda_{max} = 2 \cdot d \cdot n_{pSi},$$

where, d and n_{pSi} are the thickness and the refractive index of the porous silicon layer respectively, λ_{max} and N are the wavelength and an integer number of corresponding interference maximum respectively.

n_{pSi} was estimated by using the following equation:

$$N_i - N_j = 2dn_{pSi}\left(\frac{1}{\lambda max_i} - \frac{1}{\lambda max_j}\right)$$

2.2. Porosity determination

The average effective refractive index of porous Si can be expressed as

$$n_{PS}^{2/3} = f_{Si} n_{Si}^{2/3} + f_m n_m^{2/3},$$

where f_{Si} is the volume fraction occupied by nanocrystallites, and f_m – the medium (air or hexadecane) volume fraction. The condition $\sum_i f_i$ =1 should be satisfied.

f_{air} corresponds to the ***porosity level*** of porous Si.
From $n_{PS-hex}^{2/3}$ the ***hexadecane volume fraction*** f_{hex} was estimated.
The ***hexadecane filling factor*** (%) was calculated as a ratio between f_{hex} and f_{air}.
In the present work, n_{Si} = 3.4, n_{air}=1 and n_{hex}=1.64 were taken.

3. Local temperature measurements from Raman experiments

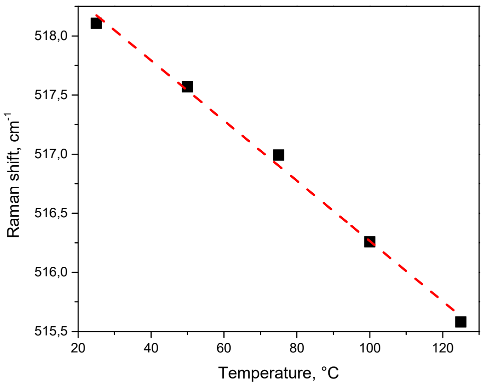

Figure S4. Correlation between Raman peak spectral position and sample temperature.
